# Rift Valley fever and invisible women

**DOI:** 10.1017/one.2025.10005

**Published:** 2025-06-04

**Authors:** Luke O’Neill, Simon Gubbins, Christian Reynolds, Kyriaki Giorgakoudi, Georgina Limon

**Affiliations:** 1 HSRM Department, School of Health and Psychological Sciences, City University of London, London, UK; 2 The Pirbright Institute, Pirbright, UK

**Keywords:** Rift Valley fever, one health, gender, disease surveillance, epidemiology

## Abstract

Public health interventions often neglect gender disparities. This perspective paper highlights the gendered risks using Rift Valley fever (RVF), a vector-borne zoonotic disease, as a case study, and discuss how gender inequality in RVF disease surveillance and control might impact women’s health. Most of the literature focuses on RVF exposure in males due to certain occupational roles being male dominated and neglects women’s varied responsibilities in livestock care. RVF-focused studies often lack sex-aggregated data, hindering our understanding of the gendered differences in RVF risk. Social and cultural norms limit women’s autonomy in livestock ownership, vaccination decisions and healthcare access. Therefore, there is a lack of gender-based policy for the prevention and control of RVF. To tackle the issues of gender inequality in disease surveillance and control, we need to integrate gendered considerations into RVF research design and analysis. This can lead to development of genderresponsive interventions for improved knowledge dissemination and access to veterinary care for women livestock keepers. Intervention programmes involving women (such as the We Rear Programme) have led to positive changes in social and cultural norms, resulting in greater access to markets and veterinary care for female farmers. Gender inequality in RVF disease surveillance compromises women’s health and the health of their livestock. Urgent action is required to bridge the knowledge gaps highlighted in this paper and develop equitable interventions for a One Health approach to the control of RVF.

## Introduction

Livestock are of vital importance to communities globally and are the primary income for approximately 70% of the 1.4 billion extremely poor. Of these it is estimated 600 million subsistence livestock farmers globally and approximately two thirds of these are women (MacVicar, 2020). It has been estimated that women contribute 40% of the agricultural labour force in Africa, though this is much higher in some countries, such as in Tanzania where women are thought to contribute 53% of the agricultural labour force (Palacios-Lopez et al., 2017). Gender inequality within the wider food system is estimated to be responsible for a loss of 11% of Africa’s total wealth, and livestock plays a crucial role in rural women’s lives (Breisinger et al., 2023). For subsistence farmers livestock are critical for their survival because livestock generate income, a store of wealth and provide nutritional security. Women in particular face many challenges, including lack of access to agricultural extension services, aid, markets, and smallholder-focused policies (Gannaway et al., 2022; E. Mutua et al., 2019). These gendered disparities in access and support also mean that women are more vulnerable to shocks that affect livestock health and productivity.

Despite the economic importance of livestock, many rural subsistence farmers do not reach maximum productivity due to high mortality and morbidity rates due to infectious disease epidemics (Mukamana et al., 2022), such as Rift Valley Fever. Rift Valley fever (RVF) is a zoonotic vector-borne disease that has severe economic impacts, affects livestock productivity and survival and is a threat to human health (Nanyingi et al., 2015; Clark et al., 2018). RVF can cause abortion storms and high mortality rates in livestock (Himeidan, 2016; Clark et al., 2018; Wright et al., 2019; McMillen and Hartman, 2021), leading to major economic impacts felt by farmers. Abortion storms refer to the sudden increase of abortions within a herd due to disease. Spillover to humans can occur via mosquito bites or close contact with infected materials, such as aerosol spray of blood or ingestion of unpasteurised milk (Clark et al., 2018; Wright et al., 2019). In human’s symptoms are often non-specific and can lead to misdiagnosis. A small proportion of cases progress onto the haemorrhagic phase of the disease, which has a significantly higher fatality rate (Javelle et al., 2020; Bron et al., 2021; Chambaro et al., 2022).

Given women’s vital but often under-recognised roles in livestock production, they are disproportionately affected by disease outbreaks (Mukamana et al., 2022; Byers et al., 2025). Yet the gendered impacts of livestock diseases remain understudied. This is especially concerning for diseases like RVF, which, impacts livestock productivity but also threatens human health, livelihoods, and food security. Understanding how these impacts differ by gender is critical to designing equitable and effective public health and veterinary responses.

The aim of this perspective paper is to use Rift Valley fever as a case study to explore how gender inequality in relation to infectious diseases poses obstacles to the safety and wellbeing of women. It extends discussion of the results on gender identified as part of a rapid review of the literature on the socioeconomic impacts of RVF (O’Neill et al., 2024). To complement this previous academic literature review we searched the Overton policy database (https://overton.io/) and the Lens database to identify additional grey literature and policy reports. Neither search identified any relevant policy or civil society documents that explicitly mentioned these issues.

### Gender disparity of exposure in occupational health

Epidemiological studies of RVF generally focus on animal infections and individuals in close contact with animals. Our previous work found no study which directly investigated the risks or impacts associated to RVF and women (O’Neill et al., 2024). Of the 17 epidemiological studies (out of 93 studies identified in our previous work) (Table 1), 11 studies had a bias towards male participants with an average of 67% male participants (range 57%–93% male). Women are grouped together under the occupation of housewife. As women have varied responsibilities, beyond household duties, including preparation of food/ animal products, tending to the young and sick livestock, and milking responsibilities (E. Mutua et al., 2019; Nyangau et al., 2021), this classification makes it difficult to assess their risk of exposure to RVF.

Previous studies have suggested that men, especially in pastoralist communities, are at a greater risk of RVFV exposure due to the extended periods of time they spend moving their livestock (cattle) between pasture, compared to women and other occupations (Affognon et al., 2017; A. Heinrich et al., 2012; Archer et al., 2013; LaBeaud et al., 2015; Ahmed et al., 2018; Bron et al., 2021; E. N. Mutua et al., 2017). Other occupations, which are generally male dominated, such as butchers and abattoir workers, are at increased risk of RVFV exposure through close contact with animal blood and bodily fluids (Heinrich et al., 2012; Archer et al., 2013; Nanyingi et al., 2015; Van Vuren et al., 2018; Msimang et al., 2019; Bron et al., 2021).

However, this assumed increased risk of exposure is not necessarily supported by seroprevalence studies, Of the six seroprevalence studies identified in our earlier research (Table 1), half (3/6) did not provide sex aggregated data for occupation. Two found no statistically significant difference in seropositivity rates between males and females and only one found males are more likely to be RVF seropositive. If studies are demonstrating no statistically significant difference in seropositivity levels between men and women, are we as researchers inadvertently reinforcing gender inequality within epidemiology studies by maintaining the narrative of men are at greater risk of RVF exposure? It is critical that gender is incorporated into epidemiological research to ensure both men and women are actively included in the planning and design of interventions. Only then can we achieve a truly One Health approach – one that develops balanced, gender-responsive strategies integrating human, animal and environmental health in RVF control and prevention.

### Gender disparities in ownership, prevention, and treatment of livestock

In many societies women face limited control over their income, ownership of higher value livestock, access to and control of productive resources, and ownership and access to land (Fischer and Qaim, 2012; Tavenner and Crane, 2018; Acosta et al., 2022; Gannaway et al., 2022; Mukamana et al., 2022; Byers et al., 2025). This is because of sociocultural, religious and institutional norms in RVF endemic countries. It must be noted that norms differ between communities and countries. A study in Rwanda reported the main barriers to women entering the livestock RVF vaccine value chain were laws and regulations, access to resources including credit, vaccines and infrastructure, cultural norms and gender stereotyping, and lastly weakness with vaccine distribution and training opportunities (Gannaway et al., 2022). These structural barriers restrict women’s autonomy of choice to protect themselves by vaccinating their livestock and highlights the clear disadvantage to women of sociocultural norms and the male-dominated design of the vaccine chain.

Women tend to own lower-value livestock, such as poultry and goats. In the context of RVF the main susceptible livestock species are cattle, sheep and goats, with sheep being the most susceptible. However, national vaccination programmes tend to focus on cattle, even though in the case of RVF sheep and goats are more susceptible compared to cattle (Acosta et al., 2022; Gannaway et al., 2022; Mukamana et al., 2022; Tukahirwa et al., 2023; Byers et al., 2025). Larger herd sizes are also prioritised for vaccination, excluding small herds often owned by women and other smallholders (Acosta et al., 2022; Tukahirwa et al., 2023). Consequently, in an RVF outbreak, sheep and goats owned by women are at risk and so is their income and access to food especially animal source protein.

As part of the invisible work women and children do in livestock rearing, women are more likely to take care of sick livestock, increasing their risk of RVF exposure (Miller, 2011; Breisinger et al., 2023). A study in Uganda reported women have excessive workloads completing more daily tasks in livestock production as compared to men, resulting in less time to tend to their own animals or attend educational programmes (Tukahirwa et al., 2023). Although women spend more time compared to men with animals, women are restricted in their autonomy on treating sick animals and vaccination of livestock. For example, a study in Kenya and Uganda has reported that even when women are head of households, they are still required to consult male family members regarding treatment of sick livestock (E. Mutua et al., 2024). These cultural and institutional norms not only limit women’s autonomy in livestock care but also influence their ability to engage in critical health interventions, such as vaccination.

In an attempt to address gendered barriers and increase vaccine uptake, a research team from ILRI, ran RVF vaccination campaigns in Kenya using a gender-based approach (Campbell, 2023). The modifications to the vaccination campaign included hiring women champions, working with community disease reporters and local leaders to ensure the correct messaging of the campaign was conveyed and providing facilities to make it easier for women to control their herds and prevent animal injury. Although preliminary data suggest that the intervention (with gender modifications) performed no better than the control group (without gender modifications), the impact indicators used were purely quantitative, limiting the ability to capture the full scope of the intervention’s effects. Logistical challenges were identified as potential reasons for the lack of positive differences in the intervention group, which included delays in vaccine delivery affecting only the intervention group, and the vaccination campaign occurred at the same time than most animals were pregnant, Nonetheless, lessons learned can inform future vaccination campaigns. Indeed, other studies have shown that reducing gendered barriers in the livestock sector increases women’s access to vaccines (McKune et al., 2021; Serra et al., 2022; Njiru et al., 2024).

Although this has not yet been translated into formal policy, the Kenyan Government has held workshops in how to consider gendered barriers to vaccine uptake for their RVF contingency plan (Bett, 2022; Campbell, 2023; Tramsen, 2023). At the time of writing (April 2025) the contingency plan is being finalised and has not been published. Other endemic countries, such as Tanzania, are also developing One Health preparedness plans for RVF. Now is the perfect time to acknowledge gender inequality and incorporate gender responsive interventions that will better target animal, environmental and the health of men, women and children in an equitable and sustainable fashion.

#### Gender disparity in knowledge of RVF

Knowledge of disease is of significant importance to reducing exposure and transmission, as a lack of knowledge can increase unsafe farming practices (Alemayehu et al., 2021). However, it has been reported increased knowledge does not always lead to good farming practices (Alhaji et al., 2018; Etter et al., 2022; Ahmed et al., 2025). Eight Knowledge, Attitudes and Practices (KAP) studies were identified in our previous research (Table 1). Six (out of eight) KAP studies were biased towards male participants (range 67%–83%). However, it is difficult to draw direct comparisons between the studies as they collected different information regarding knowledge of RVF. Moreover, KAP scores were not sex disaggregated in any of the studies, so it is not possible to distinguish if there was a knowledge-gap between men and women.

Female farmers are disadvantaged due to lack of relatable information (prevention and control) regarding RVF, and lack of access to this information (Gannaway et al., 2022). This is partly because women are not permitted to attend educational programmes if they are led by men for sociocultural and sometimes religious reasons. As a result, women have restricted access to vital information and education programmes regarding transmission, control and prevention of RVF (Njuki and Sanginga, 2013). Other examples of dissemination of information to the public, for example posters in public places (Mutua et al., 2019), are restrictive for women because of their domestic roles (Namatovu et al., 2021). Many individuals in rural pastoral communities have limited or no education, with a high rate of illiteracy. For example, it has been reported the Maasai have the highest illiteracy rates (75%) (Pesambili, 2020). This is a stark comparison to the estimated illiteracy rates of the continent of Africa which is estimated to be 33% (Statista, 2022; Mutua et al., 2019; Namatovu et al., 2021). Access to this information is therefore limited to men who can read and are attending public spaces (UNESCO Institute for Statistics, 2019). Radios are also often used to disseminate information (Mutua et al., 2019), however these are more typically used by men, and so again leaves women to rely on their male counterparts to relay the information (Namatovu et al., 2021).

Male dominated research teams are a further barrier for women to enter the livestock value chain, this further compounds the barriers discussed above. A greater inclusion of female researchers would enable more women to attend KAP studies and educational campaigns. Greater attendance of women at these events will enable them to have a greater influence on research agendas, put across their point of view and challenges faced, all of which may not be considered at a male dominated event. Empowering women through female led educational programmes will result in women gaining a greater understanding of RVF and reducing their risk of exposure (Namatovu et al., 2021).

### Maternal care

The risks posed by RVF to pregnant women are poorly understood, however, existing evidence suggests that urgent research is required to fill this knowledge gap and support the design of targeted policies to protect pregnant women during RVF outbreaks (Arishi et al., 2006; Adam and Karsany, 2008; Baudin et al., 2016; McMillen and Hartman, 2021). To our knowledge, there is no specific national policies that address RVF and maternal health services. Our previous research (O’Neill et al., 2024) and the searches of grey literature and policy documents (Overton and Lens databases) did not find any national or global level policy documents of RVF and pregnant women.

In livestock, RVF is known to cause abortion storms during outbreaks. In fact, abortion storms in livestock are often considered the first indicators of RVF outbreaks in endemic countries (McMillen and Hartman, 2021). Despite this well documented phenomenon, the potential for RVF to lead to miscarriages and other complications in pregnant women is not well understood.

Only a few studies have attempted to explore RVF related pregnancy outcomes in women. One study sampled the seroprevalence of three groups (45 women who aborted pre-outbreak; 51 women who aborted during the outbreak; and 115 randomly selected male and females from local villages) as a proxy for RVF abortions. No significant difference was seen between the three groups, with seroprevalences of 31%, 28% and 33%, respectively (Abdel-Aziz et al., 1980). Another study found a significantly higher rate of still births for RVF positive mothers (15%; 10/65) as compared to RVF negative mothers (6%; 209/ 3124) (Niklasson et al., 1987). Both studies called for larger studies to be conducted to gain a greater understanding of RVF and pregnant women, but this call has largely been unanswered.

More recent evidence of vertical transmission (transmission of RVF from mother to foetus) of RVF in pregnant women has been reported in Saudi Arabia in 2000 (Arishi et al., 2006) and in Sudan in 2007 and 2011 (Adam and Karsany, 2008; Baudin et al., 2016). The first report was in Saudi Arabia during the outbreak in 2000, where a five-day old infant was admitted to hospital with respiratory issues and died two days later. It was later found that four days prior to the delivery, the mother developed RVF-like symptoms after being in contact with sick or aborting animals during the RVF outbreak (Arishi et al., 2006). The first report in Sudan included a pregnant woman who was hospitalised with RVF symptoms and was later diagnosed with RVF. The child was born with an enlarged spleen, liver and was clinically diagnosed with jaundice (Adam and Karsany, 2008). The second report in Sudan arose from a study conducted in 2011, where 28 out of 130 pregnant women (18%) were positive for RVF infection. Of these 28 women, 54% had a miscarriage compared to 12% of women who were RVF negative. Patients positive for RVF also had higher rates of bleeding, joint pain and malaise. The same Sundanese study reported vertical transmission in women (Baudin et al., 2016). Therefore, urgent research is required to gain a greater understanding of the risks related to RVF and pregnant women.

Despite these findings, the relationship between RVF and pregnancy complications in women remains severely underexplored. More robust data and research is urgently required to understand the full extent of the risks of RVF poses to pregnant women and to aide in the development of policies that ensure maternal health protected in future RVF outbreaks.

## Conclusion

Gender inequality in RVF disease surveillance and control poses a significant threat to women’s wellbeing and livelihoods. This paper uses RVF as a case study but has also highlighted inequality using examples from other diseases. It is imperative that we acknowledge and tackle gender inequalities so that communities, public health, and veterinary systems are better prepared to respond to outbreaks in the future.

Despite an increasing frequency of RVF outbreaks, current surveillance efforts often overlook gender-specific interventions. It is evident that there is a clear knowledge gap in our understanding of transmission and impact on women. Women are also disadvantaged regarding access to knowledge and practices to prevent RVF because they do not have access to the relevant information.

Women’s participation in the agricultural sector has been widely documented, but it is critical for more gendered data on the roles of women in different contexts agricultural, livestock and vaccine value chains. This will ensure we build a greater understanding the transmission dynamics of both men and women. By incorporating gender-sensitive approaches in study design, data collection and analysis, we can target interventions and improve the effectiveness of RVF prevention and control measures for all populations.

## Figures and Tables

**Table 1:** Presents the participatory studies included in the socio-economic impact of Rift Valley fever: a rapid review and discussed in this perspective paper

Author	Study type	Total number of participants	Gender breakdown	
			Male (%)	Female (%)
Nma Bida Alhaji	KAP^1^	403	81.9	18.1
Msimang	Seroprevalence	684	93	7
Kainga	KAP	400	67.25	32.75
LaBeaud	Seroprevalence	164	48	52
Woods	Case report	77	57	43
Alhaji	KAP	389	74	26
Mutua	KAP	560	47.5	52.5
Hassan	KAP	235	47	53
Affognon	KAP	698	73	27
Ahmed	Seroprevalence	751	41.5	58.5
Mutua	Gendered barriers	645	49.9	50.1
Archer	Seroprevalence	302	87	13
Boushab	Seroprevalence	31	74	26
Nyangau	KAP	629	82	18
Ngoshe	KAP	504	82.5	17.5
Wanyoike	Willingness to pay	326	48	52
Masemola	Willingness to pay	126	75	25
Total		6924	67	33

1 KAP – knowledge, attitudes and practices.

## Data Availability

Data available within the article or its supplementary materials – “The authors confirm that the data supporting the findings of this study are available within the article.”
